# Awareness and knowledge of heterozygous familial hypercholesterolemia among Serbian pediatricians

**DOI:** 10.3389/fped.2023.1096478

**Published:** 2023-02-07

**Authors:** Ljiljana Bjelakovic, Lazar Stosic, Aleksandra Klisic, Marko Jovic, Sanja Stankovic, Aleksandra Stankovic, Sasa Pantelic, Danijela Zivkovic, Vladimir Vukovic, Bojko Bjelakovic

**Affiliations:** ^1^ Department of Medical Science, Faculty of Sport and Physical Education, University of Nis, Nis, Serbia; ^2^ Faculty of Medicine, University of Nis, Nis, Serbia; ^3^ Primary Health Care Center, University of Montenegro-Faculty of Medicine, Podgorica, Montenegro; ^4^ Faculty of Medicine, Institute of Biochemistry, University of Kragujevac, Kragujevac, Serbia; ^5^ Center for Disease Control and Prevention, Institute of Public Health of Vojvodina, Novi Sad, Serbia; ^6^ Department of Epidemiology, Faculty of Medicine, University of Novi Sad, Novi Sad, Serbia; ^7^ Clinic of Pediatrics, Clinical Center, Nis, Serbia

**Keywords:** familial hypercholesterolemia, pediatricians, questionnaire, awareness, knowledge, electronic questionnaire survey

## Abstract

**Objective:**

Published reports describing awareness and knowledge of familial hypercholesterolemia (FH) among pediatricians are few and differ considerably across countries. We aimed to assess awareness and knowledge of the FH among pediatricians in Serbia.

**Methods:**

A web-based cross-sectional study using a self-designed questionnaire was conducted during the annual congress of the Serbian Association of Preventive Pediatrics in 2020.

**Results:**

A total of 141 pediatricians completed the questionnaire (response rate 16.1%). Overall, 91% of participants have knowledge about genetic inheritance of FH, 84.3% were aware of long-term health risks of FH, 77% were familiar with normal cholesterol values in children and 71% knew the FH prevalence in the general population. On the other hand, only 36.8% declared that they were familiar with international guidelines for FH drug treatment and only 26.2% declared to have patients with FH.

**Conclusion:**

There is a substantial lack of practical clinical knowledge among Serbian pediatricians on managing children with FH. In addition, an extremely low questionnaire response rate (16.1%) suggests that most pediatricians are not aware of the clinical importance of FH in childhood.

## Introduction

Heterozygous familial hypercholesterolemia (FH) is the most common genetic metabolic lipid disorder, characterized by elevated low-density lipoprotein cholesterol (LDL-C) levels from birth and a significantly higher risk of developing premature atherosclerotic cardiovascular disease (ASCVD) in early and middle adulthood (1, 2).

Nevertheless, there is a considerable lack of knowledge among pediatricians as well as general practitioners regarding potential short- and long-term health risks of FH, in most countries (3).

Based on theoretically estimated prevalence (1/200–1/500), between 14 and 34 million individuals worldwide have FH, but less than 1% are diagnosed in most countries (4). As a result, treatment of these patients still differs considerably across the global healthcare systems and FH is considered the most underdiagnosed and undertreated metabolic disease.

The scarcity of FH cases reported in Serbia is likely indicative of a lack of awareness of this common clinical entity among healthcare workers, including pediatricians, who are particularly important for timely diagnosis and treatment of FH (5). To our knowledge, to date there have been no published studies that have evaluated knowledge and awareness of FH among pediatricians, both at a medical conference, web-based survey or *via* phone/letter/email-sent questionnaire.

According to the screen Pro FH project, only 3.9% (a total of 900 patients with FH) have been recognized in the Serbian database, based on the most recent FH estimated prevalence of 1:311–1:313 and estimated 7.2 million inhabitants in Serbia (6, 7).

The aim of this study was to assess the level of knowledge and awareness of FH among Serbian pediatricians.

## Methods

A web-based cross-sectional study was conducted *via* a short electronic questionnaire (Mentimeter, Stockholm, Sweden) at the annual online meeting of the Association of Preventive Pediatrics of Serbia (September 2020, Nis, Serbia).

All registered meeting attendees (a total of 857) received the information of the objective of the study through direct e-mail and were asked to complete the questionnaire using Mentimeter software in the next five days, comprising 6 questions as follows: (1) Is FH a hereditary disease? (2) Are you aware of long-term adverse health effects of FH? (3) Do you know the reference values of cholesterol to establish the diagnosis of FH in children? (4) Do you know the prevalence of FH in general population? (5) Are you familiar with the international guidelines for drug treatment of FH? (6) Do you have cases with FH? We also asked attendees about the duration of their medical practice.

The data was analyzed using the Statistical Package for Social Sciences (IBM SPSS Statistics version 21). Descriptive statistics was used to summarize the collected data. The chi-square test was done to determine the difference in obtained responses to each question between pediatricians with ≤5 years and >5 years in medical practice.

## Results

**Figure 1:**
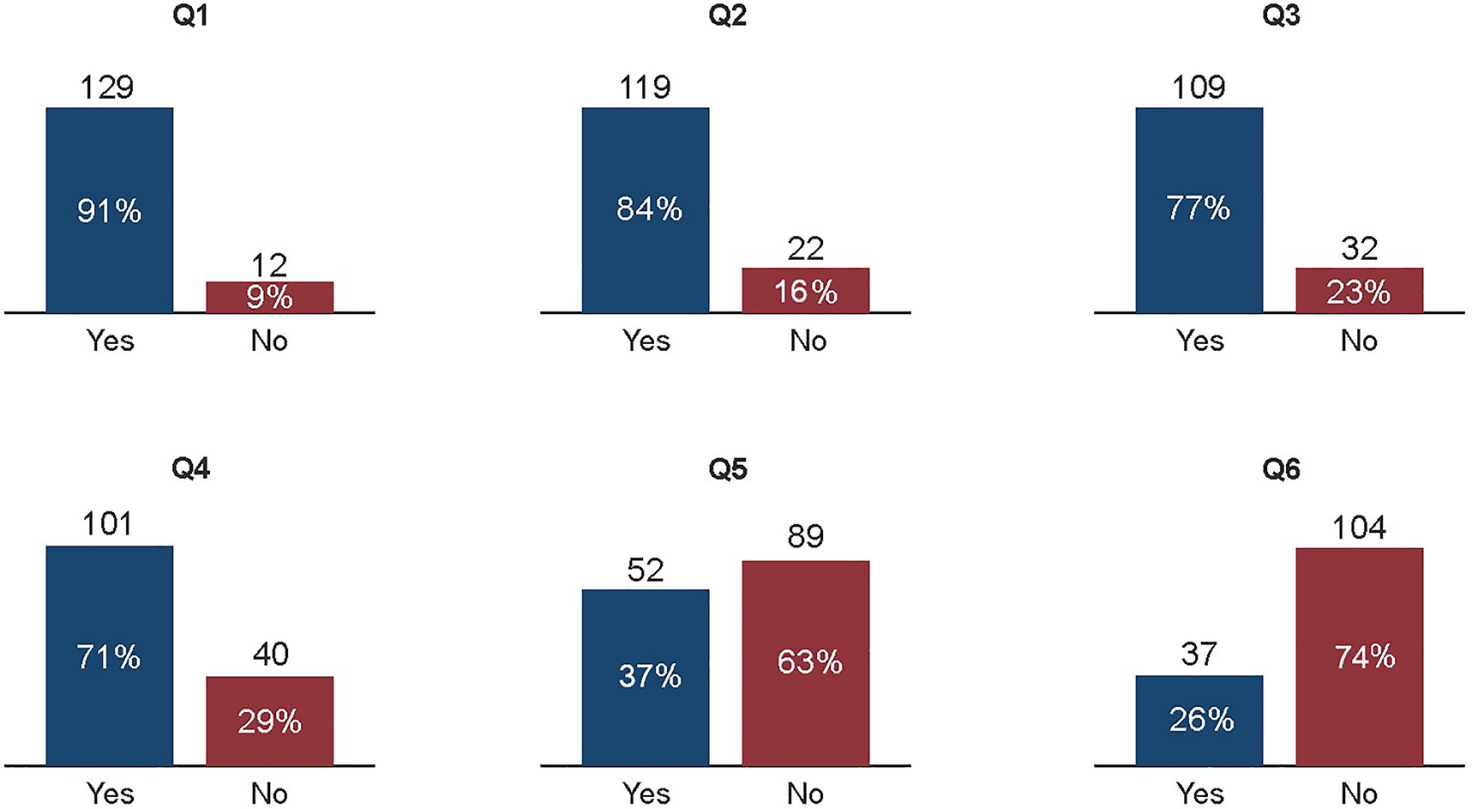
Graphic summary of the collected data. Q, Question. (Q1) Is FH a hereditary disease? (Q2) Are you aware of long-term adverse health effects of FH? (Q3) Do you know the reference values of cholesterol needed to establish the diagnosisof FH in children? (Q4) Do you know the prevalence of FH in the general population? (Q5) Are you familiar with the international guidelines for drug treatment of FH? (Q6) Do you have cases with FH?

A total of 141 pediatricians completed the questionnaire (response rate 16.1%). Majority of the participants (84.5%) were practicing pediatrics for 5 or more years and there was no difference in question responses related to years of practice. Overall, 91% participants have knowledge about genetic inheritance of FH, 84.3% were aware about long-term health risks of FH, 77% were familiar with normal cholesterol values in children and 71% knew the FH prevalence. On the other hand, only 36.8% participants declared that they are familiar with the international guidelines regarding FH management and only 26.2% declared to have patients with FH. Figure 1 presents graphic summary of collected data.

## Discussion

As previously reported, heterozygous FH is the most common genetic lipid disorder related to higher risk of premature ASCVD disease (4). Hence, timely detection, early and adequate treatment of patients suffering from FH, are essential in preventing cardiovascular events later in life (8). However, absence of the lipid clinics, national registries and initiatives on screening for FH in many countries worldwide, including Serbia, indicate important shortcomings of healthcare and medical education institutions which are directly and reciprocally linked (9).

To our knowledge there have been no published reports on awareness and knowledge of heterozygous FH among pediatricians (10). In addition, only a few studies have investigated this topic in physicians working with adult patients, mainly in family medicine and general practitioners (11, 12).

Our findings agree with most of the previous studies conducted on general physicians and family physicians, demonstrating very low awareness and practical knowledge of heterozygous FH among them (11, 12).

Although most pediatricians in our study have expressed excellent theoretical knowledge on genetic inheritance (91%), reference cholesterol values (77%) and prevalence (71%) of FH, important deficits were identified in their practical experience and knowledge in managing children with FH. Only 36.8% participants were familiar with international guidelines for drug treatment of FH children, which is in agreement with results of study published by Dixon et al. (10). Besides, only 26.2% declared to have patients (children), with FH which is similar to the results of a study conducted in the UK by Kwok et al., thus confirming common underdiagnosis of this condition in clinical practice (13).

Surprisingly, most pediatricians in our study were familiar with the long-term health risks (84.3%) which is markedly more than reported in the previous studies among the general physicians (10–12). In a study of Pang et al. only 29% of the participants recognized the increased cardiovascular risk while only 14% were correct in estimating increased risk of heart disease in a UK study of Kwok et al. (13, 14).

The extremely low questionnaire response rate (16.1%) suggests that most pediatricians in Serbia are not aware of the clinical importance of FH in childhood which is not quite different from the results in British study, reporting response rate of 31% (13). There is also a possibility of selection bias due to the fact that some pediatricians missed to receive our mail and to complete it on time. Also, some of them might lack of interest or needed extended response time.

To summarize, our pioneer attempt to get insight on this topic in Serbia indicates substantial lack of practical clinical knowledge among Serbian pediatricians on managing children with FH, which underscores the need for increased pediatric education regarding FH. Our results also implicate the importance of more effective relations between European and national authorities to prioritize and actively promote the need for early diagnosis of FH.

Since the cardiovascular morbidity and mortality rate in Serbia is among the highest in Europe, there is a need to improve current educational medical programs to adequately solve practical clinical problems of FH diagnoses and management, including a mechanism for identifying patients (children) with FH (15).

Limitations of this study are the relatively small sample size and low response rate (16.1%) which could lead to potential selection bias.

## Data Availability

The raw data supporting the conclusions of this article will be made available by the authors, upon the reasonable request.
